# Effectiveness of an intervention designed to optimize statins use: a primary prevention randomized clinical trial

**DOI:** 10.1186/1471-2296-15-135

**Published:** 2014-07-15

**Authors:** Mireia Fàbregas, Irene Berges, Francesc Fina, Eduardo Hermosilla, Ermengol Coma, Leonardo Méndez, Manuel Medina, Sebastià Calero, Elena Serrano, Rosa Morros, Mònica Monteagudo, Bonaventura Bolíbar

**Affiliations:** 1 ABS La Marina, SAP Esquerra, Institut Català de la Salut, Barcelona, Spain; 2 Institut Universitari d’Investigació en Atenció Primària Jordi Gol (IDIAP Jordi Gol), Barcelona, Spain; 3 Hospital Parc Taulí, Sabadell, Spain; 4 Sistemes d’Informació d’Atenció Primària (SISAP) – Sistema d’Informació per al Desenvolupament de la Investigació en Atenció Primària (SIDIAP), Institut Català de la Salut, Institut Universitari d’Investigació en Atenció Primària Jordi Gol (IDIAP Jordi Gol), Barcelona, Spain; 5 Àrea de Desenvolupament Clínic, Direcció Adjunta d’Afers Assistencials, Institut Català de la Salut, Institut Universitari d'investigació en Atenció Primària Jordi Gol (IDIAP Jordi Gol), Barcelona, Spain; 6 Centre d’Atenció Primària Baix a Mar, Consell comarcal del Garraf, Vilanova i la Geltrú, Barcelona, Spain; 7 Universitat Autònoma de Barcelona, Cerdanyola del Vallès, Spain

**Keywords:** Primary health care, Lipid-lowering therapy, Prescriptions, Prescription adequacy, Primary prevention, Electronic health records, Clinical practice guidelines, Clinical trial, Cardiovascular disease

## Abstract

**Background:**

Although hypercholesterolemia is considered a cardiovascular risk factor, in isolation it is not necessarily sufficient cause for a cardiovascular event. To improve event prediction, cardiovascular risk calculators have been developed; the REGICOR calculator has been validated for use in our population. The objective of this project is to develop an intervention with general practitioners (GPs) and evaluate its impact on prescription adequacy of cholesterol-lowering drugs in primary prevention of cardiovascular disease and in controlling the costs associated with this disease.

**Methods:**

This nonblinded, cluster-randomized clinical trial analyzes data from primary care electronic medical records (ECAP) and other databases. Inclusion criteria are patients aged 35 to 74 years with no known cardiovascular disease and a new prescription for cholesterol-lowering drugs during the 2-year study period. Dependent variables include the following: RETIRA, defined as new cholesterol-lowering drugs initiated during the year preceding the intervention, considered inadequate, and withdrawn during the study period; EVITA, defined as new cholesterol-lowering drugs initiated during the study period and considered inadequate; COST, defined as the total cost of inadequate new treatments prescribed; and REGISTER, defined as the recording of cardiovascular risk factors. Independent variables include the GP’s quality-of-care indicators and randomly assigned study group (intervention vs control), patient demographics, and clinical variables. Aggregated descriptive analysis will be done at the GP level and multilevel analysis will be performed to estimate the intervention effect, adjusted for individual and GP variables.

**Discussion:**

The study objective is to generate evidence about the effectiveness of implementing feedback information programs directed to GPs in the context of Primary Care. The goal is to improve the prescription adequacy of lipid-lowering therapies for primary prevention.

**Trial registration:**

ClinicalTrials.gov Identifier: NCT01997671. November 28, 2013.

## Background

Primary care medicine has included a focus on prevention for decades, but the possible side effects of preventive treatments were unknown or underemphasized. More recently, these possible risks have taken on greater importance in clinical practice [1,2], making the population aware of the potential harms associated with prevention efforts.

### Hypercholesterolemia as a risk factor

The treatment of hypercholesterolemia, a risk factor for cardiovascular disease, is a well-established preventive action; its association with increased incidence of ischemic heart disease has been described together with smoking, hypertension, and diabetes as cardiovascular risk factors (CVRF). In the Seven Country Study [3], total cholesterol of 200 mg/dl was associated with a cardiovascular mortality rate of 15% in the northern countries of Europe, compared to 3% in the south (results adjusted for age and other CVRF). This implies that, in isolation, the role of hypercholesterolemia in the incidence of cardiovascular events is neither essential (cardiovascular events occur in the absence of hypercholesterolemia) nor sufficient (most patients with high cholesterol do not have cardiovascular events) [4].

For this reason, the various models of cardiovascular risk calculation incorporate the presence of other risk factors and are not based exclusively on hypercholesterolemia [5]. Therefore, the calculation of cardiovascular risk is a tool for classifying patients according to their risk and contributes to decisions on hypercholesterolemia treatment, focusing on decreasing the incidence and mortality rates associated with cardiovascular disease rather than the potential benefits of intermediate outcomes such as lower levels of low-density lipoproteins (LDL) and total cholesterol [6].

### Repercussions of inadequate use of cholesterol-lowering drugs in primary prevention

Pharmacological treatment of hypercholesterolemia is indicated in two different groups of patients: those with and without cardiovascular disease (primary and secondary prevention, respectively). At present, there is no question about the indications and benefits of this therapy in secondary prevention [7]. Achieving cholesterol reduction objectives is usually included in evaluations of clinical management. In our context, indicators of LDL cholesterol control in patients with cerebrovascular disease and ischemic heart disease are available dating back to 2007, when the electronic medical records system was implemented. Data include monthly monitoring and feedback and asynchronous reminders to all primary care centers (PCC) within the Catalan Institute of Health (ICS: Institut Català de Salut) system. Since January 2009, secondary prevention results (adequate LDL control) have improved 21.87% for cerebrovascular disease and 15.97% for ischemic heart disease.

In primary prevention, on the other hand, drug therapies continue to be debated for two main reasons. First, no benefit has been demonstrated in women, elderly, and low-risk patients [8]. Second, differences between the criteria established in various guidelines and consensus documents result in a variable clinical impact with respect to the number of patients for whom treatment is indicated [9]. In contrast to secondary prevention, clinical assessments in primary prevention do not include a monitoring of treatment adequacy, despite the possible “medicalizing” impact associated with assigning a label and treatment and the potential consequences for the patient’s quality of life.

It is also unclear whether primary prevention drains resources that could be directed to other clinical areas. The increased prescription of cholesterol-lowering drugs in national health systems [10] has had a major impact on pharmaceutical expenditures; the greatest cost is for statins, as a group. Few studies have analyzed the distribution of this increased cost [11-13], but inadequate prescription of cholesterol-lowering drugs has been reported both as underutilization in high-risk patients and overutilization in low-risk patients [14].

Improvement in prescription adequacy requires that we address multiple factors, because this does not depend exclusively on improved adherence to clinical recommendations [15]. Of the factors to be considered, we would highlight two: the general practitioner (GP) faces challenges in interpreting results and recommendations from trials with methodological biases and marginal population-level benefit [8], and the influence of the pharmaceutical and food products industries is evident in the presentation of clinical trial results that emphasizes relative risk [16], with messages about the need to measure cholesterol levels and lower them with drug therapies that can prevent premature death in the healthy general population [17,18].

### Interventions to improve prescription adequacy

Several different types of intervention studies have been conducted in an effort to improve prescription adequacy in clinical practice. These can be grouped into three major types of intervention: 1) feedback or audits [19], in which information about clinical practice is *a posteriori*; 2) alerts or reminders [20], which suggest an action such as preventive care at the time of the patient visit or *a priori*; and 3) clinical decision support systems (CDSS) [21], which are more complex systems that guide practitioners and provide recommendations on clinical decisions to be made, based on information added to the clinical record.

In a 2010 review of literature on interventions designed to improve clinical practice, Cochrane concluded that feedback leads to slight or moderate improvement [20]. A recent clinical trial, better adapted to the characteristics of our environment [22], demonstrates major variations in carrying out preventive activities such as vaccinations, comparing usual practice with message alerts and alerts plus feedback. The progressive computerization of our primary care system creates a unique opportunity to develop interventions: the electronic medical records system is fully implemented in all health centers administered by the ICS and information systems are available that support the use of quality indicators and provide feedback to health care professionals –with or without economic incentives. Although these may have a moderate impact, their low cost and the possibility of adapting them to provide personalized information, whether in real time or asynchronously, offer enormous intervention potential.

For all these reasons, given the concerns generated by current statins prescription as a primary prevention strategy and the opportunity offered by information systems based on electronic medical records, this clinical trial is designed to evaluate the effectiveness of an intervention intended to improve the pharmacological approach to hypercholesterolemia in primary prevention.

### Research hypotheses

Inadequate prescription of cholesterol-lowering drugs in patients with no history of cardiovascular disease may have negative repercussions for the patient as well as for the health care system.

Implementation of an intervention designed for GPs in primary care and based on informational feedback concerning the adequacy of prescribed cholesterol-lowering drugs could strengthen their competency, their adherence to clinical practice guidelines (CPG), and overall efforts to contain health care costs.

### Objectives

#### ***Primary objective***

To evaluate the effectiveness of an intervention directed to primary care GPs, based on providing informational feedback about the adequacy of using cholesterol-lowering drugs in primary prevention, and designed to improve prescription practices according to the recommendations in clinical practice guidelines and to reduce health care costs, compared to current practice.

#### ***Secondary objectives***

1. To describe demographic and clinical characteristics of patients receiving new prescriptions for cholesterol-lowering drugs.

2. To determine the degree of adequate prescription of cholesterol-lowering drugs in patients treated for primary prevention (i.e., without previous history of cardiovascular disease).

3. To identify the factors associated with adequate prescription of cholesterol-lowering drugs in primary prevention.

4. To analyze the degree to which cardiovascular risk is recorded in electronic medical records after the intervention.

5. To determine the economic impact of the cholesterol-lowering drugs prescribed for primary prevention during the study period.

## Method

### Study design

The design is a multicenter, controlled, cluster-randomized, nonblinded clinical trial to evaluate the effectiveness of an intervention related to the prescription of cholesterol-lowering drugs in primary prevention, compared to usual clinical practice.

### Setting

All individuals attended in 283 primary care center (PCC) managed by the ICS, which cover a population of 5.8 million urban, semi-urban, and rural residents of Catalonia.

### Study population

Twelve PCC are excluded because their current participation in a different intervention could invalidate the results of this study. The research team will identify all patients aged 35 to 74 years who received a new prescription for cholesterol-lowering drugs from the remaining 271 PCC during the study period (October 1, 2011-September 30, 2013), as recorded in the electronic medical records system, known as ECAP. Exclusion criteria are any history of cardiovascular disease (ischemic heart disease, cerebrovascular accident, or peripheral artery disease) recorded in ECAP and patients for whom pharmacy records showed receipt of a cholesterol-lowering prescription drug during the 12 months preceding their inclusion in the study.

### Randomization

The unit of randomization is the PCC. This avoids a possible contamination effect if different health professionals from the same PCC were randomized to different study groups. Cluster randomization will be done by random-sized blocs of PCCs (i.e., 2,4,6,8,10), stratified in quartiles by a synthetic PCC quality care indicator, “EQA” [23]. This method will ensure that each study group has the same number of PCCs and GPs and that there is a balanced distribution of the EQA covariate between the two groups [24,25].

### Blinding

Due to the nature of the intervention, GPs in the intervention group cannot be blinded to their group assignment. However, the researcher responsible for data analysis will be blinded to study group assignments.

### Intervention design

Based on the list of patients with inclusion and exclusion criteria, the research team will analyze the adequacy of new prescriptions for cholesterol-lowering drugs, based on the recommendations contained in ICS clinical practice guidelines on cholesterol and coronary risk [26]. The flow chart for this analysis is included as Additional file 1.

All GPs assigned to the intervention group will receive informational feedback about the adequacy of cholesterol-lowering drugs prescribed for their patients. Feedback is provided as part of the ECAP system capabilities. The preparation and dissemination of intervention-related feedback will be monthly, beginning in the second year of the study (October 1, 2012-September 30, 2013). This information, available to the GPs at their convenience, will be presented in two distinct and complementary formats and updated throughout 12 consecutive months:

1. Asynchronous information (patient not present)

a) Quantitative information about the percentage of the GP’s assigned patients who have started primary cardiovascular prevention therapy with cholesterol-lowering drugs and that is considered correctly indicated according to the criteria established in the corresponding ICS clinical practice guidelines [26].

b) List of names of patients who have started primary cardiovascular prevention therapy with cholesterol-lowering drugs but do not meet the guidelines criteria.

2. Real-time information (patient present when information is presented to GP).

3. In each GP’s daily schedule, an alert symbol will indicate appointments with patients who have started statins therapy for primary cardiovascular prevention without meeting the established indications criteria. In addition, a message will be included in the ECAP system of clinical advisories. This screen, used during the patient visit, shows all active clinical advisories and alerts for that specific patient. This may include EQA information, medication safety considerations, notice of availability of test results, etc.

The GPs assigned to the control group will not receive the project’s ECAP feedback. The study algorithm is diagrammed in Additional file 2.

The research team will carry out the analysis of prescription adequacy. Implementation of ECAP feedback will be the responsibility of the SISAP team (Sistema de Informació dels Serveis d’Atenció Primària), the ICS unit that provides clinical practice information to ICS primary care professionals and administrators [26].

A pilot study was conducted with GPs selected from two primary care centers. This study ensured that data extraction and information alerts worked correctly. Data for these GPs will be excluded from the final results.

### Outcomes

New treatment with cholesterol-lowering drugs is defined as a prescription given to a patient who has not collected any such prescription from a pharmacy during the 12 months preceding study inclusion, according to national health system records.

Primary prevention is considered the absence of cardiovascular disease (ischemic heart disease, cerebrovascular accident, peripheral artery disease).

The ICS clinical practice guidelines on cholesterol and coronary risk are applied to determine prescription adequacy when cholesterol-lowering drugs are prescribed for primary prevention [26]. To operationalize the algorithm, adequate treatment is defined as meeting one or both of the following criteria:

A. At least 10% cardiovascular risk, according to REGICOR 10-year risk calculated within 12 months of treatment initiation. The REGICOR risk function has been calibrated and adapted to local incidence and distribution of risk factors in Catalonia [27]. It stratifies cardiovascular risk into 4 classifications: low (<5%), moderate (5%–9.9%), high (10%-14.9%), and very high (>15%). The ICS clinical practice guidelines recommend the use of REGICOR tables to calculate coronary risk and consider individuals with at least 10% risk of a 10-year coronary event to be candidates for more intensive interventions, which may include cholesterol-lowering drugs. Additional file 3 contains the REGICOR risk tables, adapted from the Framingham risk calculation.

B. At least 240 mg/dl LDL cholesterol recorded during the 12 months preceding treatment initiation.

#### ***Primary outcomes***

The primary study variable is improved prescription adequacy in the use of cholesterol-lowering drugs for primary prevention of cardiovascular disease according to the recommendations of ICS clinical practice guidelines, comparing the intervention group to usual clinical practice.

Two dependent variables will be analyzed to address the primary objective:

RETIRA, defined as new cholesterol-lowering drugs initiated during the year preceding the intervention, considered inadequate, and no longer prescribed after the GP receives feedback information. This variable is directly affected by the intervention because patient records included in the calculation of this variable will be precisely those that triggered the intervention feedback.

EVITA, defined as new cholesterol-lowering drugs initiated during the 12 months follow-up period and considered inadequate.

Improved prescription adequacy is defined as an increase in the RETIRA variable and decrease in the EVITA variable.

#### ***Secondary outcomes***

– To assess the recording of cardiovascular risk in electronic medical records, the REGISTER variable will be calculated: number of patients with no history of cardiovascular disease (primary prevention) treated with statins without having any record of a cardiovascular risk calculation.

– To assess the economic impact of prescriptions, the COST variable will be calculated: the total cost of all prescriptions for new cholesterol-lowering drugs in primary prevention reimbursed by the national health system that are considered inadequate during the study period.

The intervention is designed to produce an increase in the REGISTER variable and a decrease in the COST variable.

#### ***Other variables***

The primary independent variable is the GP’s assigned study group (intervention or control group).

Patient variables: age, sex, new statin prescription picked up from a pharmacy, cardiovascular risk (calculated using the REGICOR formula and recorded in ECAP), cholesterol (total, LDL and HDL), systolic and diastolic blood pressure, smoking habit, treatment group from the catalog of statins (Anatomic, Therapeutic, Chemical [ATC] code), and diagnosed cardiovascular disease, diabetes mellitus, or hypertension, according to International Classification of Diseases (ICD-10 codes), listed below:

– Myocardial infarction: I21 and subtypes, I22 and subtypes, I23 and subtypes, I25.2

– Ictus: I60 and subtypes, I62 and subtypes, I63 and subtypes I64

– Peripheral artery disease: I73, I73.8, I73.9

– Tobacco use: F17 and subtypes F17.1 – F17.9

– Diabetes Mellitus: E11 and subtypes, E12 and subtypes, E13 and subtypes, E14 and subtypes

– Arterial hypertension: I10, I15, I15.1, I15.2, I15.8, I15.9.

#### ***General practitioner variables***

– EQR: SIDIAP has developed this synthetic index showing the quality standard of each GP’s data recording in the ECAP system.

– EQPF: This ICS synthetic indicator reflects the quality (adequacy) of drug prescription for each GP.

– EQA: This ICS synthetic indicator reflects the overall quality of care provided by each GP, based on established ICS standards.

– Use of the intervention: SISAP provides a monthly summary of the number of time each GP accesses the information provided in ECAP.

– Teaching responsibilities: This variable reflects whether or not the GP works with a resident physician.

The patient and GP variables analyzed also allow a description of the use of cholesterol-lowering drugs for primary prevention and the associated factors that are relevant to the secondary objectives.

### Data collection and follow-up

Most of the study variables will be obtained from the population-based SIDIAP database (*Sistema d’Informació pel Desenvolupament d’Investigació en Atenció Primària*) [28], which is similar to other European databases such as Clinical Practice Research Database (CPRD), Quality database for ethical research (QRESEARCH), Out-Patient Pharmacy Database (PHARMO), or The Health Improvement Network (THIN). The SIDIAP database contains anonymized data from 5.8 million individual patients in the ICS electronic medical records system and from complementary sources that provide valid, reliable information for research about primary care. This database is representative of Catalonia as a whole (http://www.sidiap.org) and includes the following information:

1. ECAP data on sociodemographic variables, primary care visits, diagnoses (ICD-10 codes), clinical variables, prescriptions, immunizations, and background. ECAP implementation began in 1998 and has been used in routine practice since 2005 in all PCC managed by ICS.

2. Pharmacy records:

Pharmacy records: Information on medication dispensed in community pharmacies since 2005, including the associated cost.

3. Laboratory tests: Results are available for all tests since 2006.

Finally, to complete the identification of patients with a history of cardiovascular disease, hospital discharge data are available for all public hospitals in Catalonia (Minimum Basic DataSet).

### Sample size

The preliminary study conducted by the research team showed the availability of a sample size of approximately 60,000 new statin treatments prescribed each year. Assuming 90% strength, 5% alpha error, a 1:1 ratio of control to intervention participants, corrected by the cluster design and assuming 10% loss to follow-up, our study could detect a difference of 1% (according to the medical criteria applied by the experts on the research team) at a 90% confidence interval. Even with this strength level, a clinically relevant difference of 10% can be established.

### Statistical analysis

Initially, descriptive analysis will be completed for all study variables: mean (standard deviation) for continuous variables with a normal distribution, median (interquartile ranges) in the case of non-normal distribution and the frequency (percentage) of categorical variables. Differences between the intervention and control groups will be assessed using the χ2 or Fisher exact test for categorical variables and Student *t* for continuous variables with normal distribution, or nonparametric Mann–Whitney U test, as appropriate. Differences between means, medians, or ratios at the beginning and end of the two-year follow-up period (pre- and post-intervention) will be compared using the McNemar test, Student *t* for paired tests, or Wilcoxon test, as appropriate.

The percent change with respect to baseline value is used as a measure of the pre- and post-intervention change in categorical variables, calculated as the final percentage minus the initial percentage × 100.

The intervention effect after one year of follow-up will be evaluated using the decrease in the EVITA variable and increase in the RETIRA variable. To assess the effect of the intervention model, a multilevel logistic regression analysis will be performed, considering intervention vs control group as the independent variable. The analysis will be adjusted for potential predictive independent variables and, for those considered clinically relevant, for baseline values.

To determine the economic impact of new prescriptions of cholesterol-lowering drugs in primary prevention prescribed during the study period, a descriptive analysis of costs and resource use will be completed for each study group and the groups will be compared (discount rate: 3%). The incremental cost-effectiveness ratio will be calculated as the difference between the cost average for the 2 study groups, divided by the difference between the effects for both groups. Acceptance curves will be calculated to determine the cost-effectiveness of the intervention compared to usual practice.

Significance will be established at the 5% level. All analysis will be done using Stata/SE, version 10.1 (StataCorp).

### Research ethics

The study will follow the national and international norms defined in the Declarations of Helsinki and Tokyo, as well as the recommendations of the Clinical Research Ethics Committee (CEIC) of our research institute, the Institut Universitari d’Investigació en Atenció Primària (IDIAP) Jordi Gol. Confidentiality of personal data is guaranteed for all participants in accordance with Spanish Law (*LOPD: Ley Orgánica de Protección de Datos de Carácter Personal 15/1999, 13 December*).

The data included in the SIDIAP database are anonymized and identified only by an internal code, created when the record is created; personal identification of patients by the research team is impossible. All PCC will be informed about the study outcomes.

To further guarantee confidentiality, SIDIAP has a protocol for linking records with external databases such as the CMBD-AH, and information provided by SISAP does not contain patient-identifying data. The procedure used consistently by SISAP is that anonymized codes are assigned, which the ECAP system decodes when information is provided to professionals responsible for that patient’s care.

## Discussion

The results of this trial will provide new evidence of the effectiveness of an intervention based on clinical advice and feedback to primary care professionals via electronic medical records information. Extensive computerization of PCC records allows the creation of mechanisms and instruments to help the professional improve his or her approach to particular health problems and adherence to current clinical practice guidelines.

There are major problems with pharmacological treatment for the primary prevention of hypercholesterolemia, including a high percentage of patients being treated inappropriately under established guidelines. It is a priority to address these problems within PCCs, in order to avoid negative consequences for patients as well as for the health care system.

This trial will be conducted in a broad population of more than 5 million patients, which results in sufficient strength to obtain significant results. In addition, evaluation of the intervention will be based on an information system that has been widely studied and validated, the SIDIAP.

In any case, the study has several limitations, which we have attempted to address. One assumes that all databases (such as SIDIAP) have a certain level of underreporting on variables of interest, although this should not differ between the two randomized study groups. In our case, data recording in the electronic medical records should be reliable: most of the study variables form part of the quality indicators that are incentivized by the ICS payment systems. In addition, one of the study variables, EQR, is the level of professional quality, which allows a sub-analysis of the quality of the services provided. To avoid the loss of recorded diagnoses, any diagnosis of cardiovascular disease recorded in the electronic medical record will be complemented with hospital discharge diagnostic data. With respect to the treatment variables, the records are reliable because the data are taken from the pharmacies’ invoicing system for national health system reimbursement for the prescriptions they filled for patients.

The fact that the groups are not blinded is a limitation of the intervention, and the possibility of contamination between groups could limit the study conclusions. Nonetheless, we attempt to evaluate the effectiveness of an intervention in a real-life context linked to normal clinical practice, where this type of situation is quite frequent. Randomization by PCC rather than by individual GP reduces the probability of contamination between the study groups.

In conclusion, this study contributes new knowledge about interventions that can improve the prescription of cholesterol-lowering drugs by following the recommendations in clinical practice guidelines, and can help to reduce health care costs. We consider this study very valuable for informing health care professionals about changes that can be made, based on the information received in PCC.

The study provides an idea of usual clinical practice by primary care professionals related to the adequacy of cholesterol-lowering drug prescriptions for primary prevention. At the same time, it allows us to evaluate possible changes that could occur naturally after an intervention related to the proper prescription of cholesterol-lowering drugs. This study could have special relevance because of the enormous potential offered by the low cost of the intervention and the possibility of adapting the electronic medical records system to provide personalized information, whether in real time or asynchronously.

## Abbreviations

CDSS: Clinical Decision Support System; CEIC: Clinical Research Ethics Committee; CMBD-AH: Minimum Basic Dataset of Admissions to Hospital; CPG: Clinical practice guidelines; CPRD: Clinical Practice Research Database; CVRF: Cardiovascular risk factor; ECAP: Electronic health record system of the Institut Català de la Salut in primary care, *estació Clínica d’Atenció Primària*; EQA: Quality Care Indicator; EQPF: Prescription Quality Indicator; EQR: Quality of data recording; GP: General practitioner; HDL: High-density lipids; ICD: International Classification of Diseases; ICS: *Institut Català de la Salut*; IDIAP: Institut Universitari d’Investigació en Atenció Primària; LDL: Low-density lipids; PCC: Primary Care Center; PHARMO: Out-Patient Pharmacy Database; QRESEARCH: Quality database for ethical research; REGICOR: *Registre Gironí del Cor*; SIDIAP: Information system for research on primary care: *Sistema de Informació pel Desenvolupament d’Investigació en Atenció Primària*; SISAP: Primary care database: *Sistema d’Informació dels Serveis d’Atenció Primària*; THIN: The Health Improvement Network.

## Competing interest

This manuscript contains no information on medical devices. The authors neither received, nor will receive, individual financing of their work. The authors declare that there are no competing interests.

## Authors’ contributions

BB is the principal investigator and coordinator of the project. The study was designed by BB, MF, IN, and FF. These authors, with the help of EH, MM, SC, RM and ES, designed the intervention. EH, FF, RM were responsible for database creation and management. The intervention was implemented by FF, EH, EC, LM, SC and MM. EH performed the statistical analyses. All the authors have read and revised the various versions of the manuscript and have approved this final version.

## Pre-publication history

The pre-publication history for this paper can be accessed here:

http://www.biomedcentral.com/1471-2296/15/135/prepub

## Supplementary Material

Additional file 1 **Catalan Institute of Health Clinical Guidelines for Primary Prevention of Cardiovascular Disease **[26]**.** Click here for file

Additional file 2 Study algorithm. Click here for file

Additional file 3 Framingham-REGICOR adapted risk charts, simplified into four risk categories based on event concentration data and the 10-year risk cutoff values proposed by experts from various autonomous communities in Spain. Click here for file
