# Supplementary material for: Effectiveness of an intervention designed to optimize statins use: a primary prevention randomized clinical trial
Source: BMC Fam Pract. 2014 Jul 15;15:135. doi: 10.1186/1471-2296-15-135 (PMC4112648; doi:10.1186/1471-2296-15-135)
Supplement: Additional file 1 — Catalan Institute of Health Clinical Guidelines for Primary Prevention of Cardiovascular Disease [26]. [file 1471-2296-15-135-S1.docx]

Additional file **1: Catalan Institute of Health Clinical Guidelines for Primary Prevention of Cardiovascular Disease**^26^

Measure

Total Cholesterol (TC)*


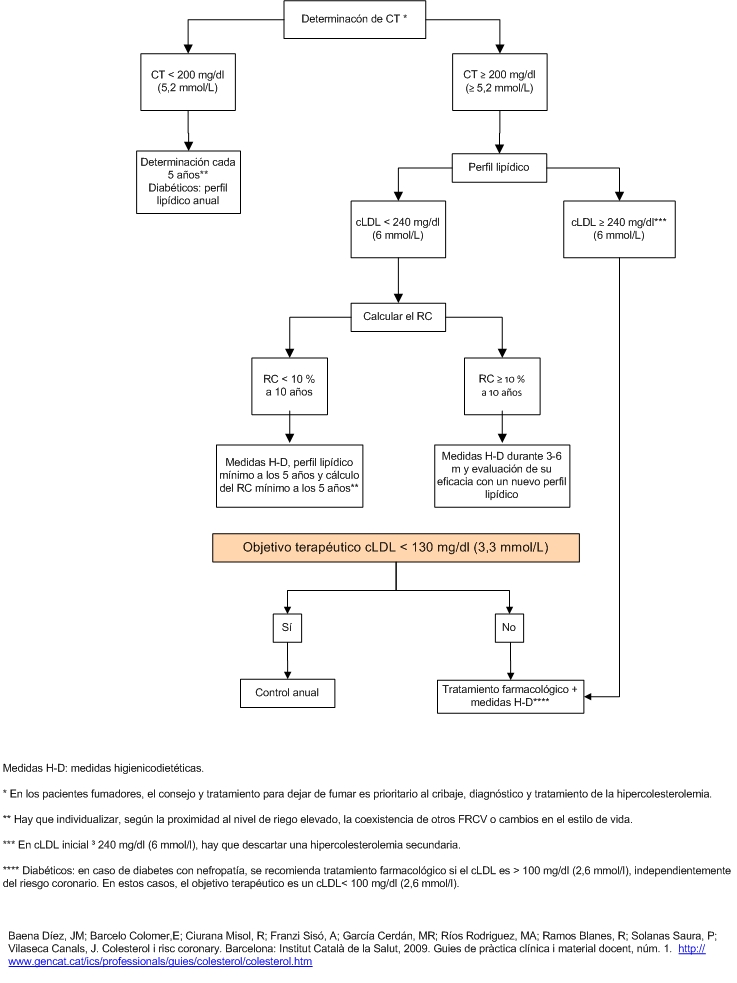


10Y CVR<10%

10Y CVR ≥10%

Calculate 10-year cardiovascular risk (10Y CVR)

No

Yes

Drug therapies +

H-D**** assessments

Treatment target: LDLc < 130 mg/dl (3.3 mmol/L)

Assess H-D, lipid profile and CVR every 5 years**

Assess H-D for 3-6 months and evaluate effectiveness with repeat lipid profile

H-D assessments: health and dietary measures

*For smokers, counseling and smoking cessation treatment is prioritized over hypercholesterolemia screening, diagnosis, and treatment.

**Individualized according to existence of other cardiovascular risk factors, depending on proximity to high-risk values and lifestyle changes.

***If initial LDLc is 240 mg/dl (6 mmol/L), secondary hypercholesterolemia must be discarded.

****Diabetics: In the presence of kidney disease, drug therapy is recommended if LDLc is > 100 mg/dl (2.6 mmol/L), independently of coronary risk. In these patients, the treatment objective is LDLc < 100 mg/dl (2.6 mmol/L).

^26^ Baena Díez, JM; Barcelo Colomer, E; Ciurana Misol, R; Franzi Sisó, A; Garcia Cerdán, MR; Ríos Rodriguez, MA; Ramos Blanes, R; Solanas Saura, P; Vilaseca Canals, J. Colesterol i risc coronari. Barcelona: Institut Català de la Salut, 2009. Guies de pràctica clínica i material docent, núm 1. <http://www.gencat.cat/cs/professionals/guies/colesterol/colesterol.htm>

LDLc ≥ 240 mg/dl

(6 mmol/L)***

TC < 200 mg/dl (5.2 mmol/L)

Annual monitoring

LDLc < 240 mg/dl

(6 mmol/L)

Lipid profile

Retest every 5 years*

* Diabetics: Annual lipid profiles

TC ≥ 200 mg/dl (5.2 mmol/L)
