# Supplementary material for: Effectiveness of an intervention designed to optimize statins use: a primary prevention randomized clinical trial
Source: BMC Fam Pract. 2014 Jul 15;15:135. doi: 10.1186/1471-2296-15-135 (PMC4112648; doi:10.1186/1471-2296-15-135)
Supplement: Additional file 2 — Study algorithm. [file 1471-2296-15-135-S2.docx]

283 PCC

Enter study:

Patients aged 35-74 years with a new prescription for cholesterol-lowering drugs during the study period (1 October 2011 - 30 September 2013), recorded in electronic medical record (eCAP)

Patients excluded due to:

- cardiovascular history

-ischemic heart disease

-cerebrovascular accident

-peripheral artery disease

- cholesterol-lowering drugs during the 12 months preceding the study

INTERVENTION

[134 PCC]

CONTROL

[137 PCC]

General practitioners (GPs) receive both real-time and asynchronous feedback about the adequacy of their patients’ cholesterol-lowering therapy from the electronic medical records system (eCAP):

- Asynchronous information (the patient is not present)

-Information related to the percentage of patients receiving the correct treatment as indicated.

-List of patient names whose treatment is incorrectly indicated.

- Real-time information (the patient is present).

-Warning if the patient is receiving incorrect treatment.

-Addition of an indication within the eCAP clinical alerts system.

12-MONTH FOLLOW-UP

-Analysis of treatment effectiveness of new hypolipidemia treatments prescribed.

Standard/normal care

Additional file 2. Study algorithm

271 PCC

12 PCC excluded due to an intervention that could invalidate study results
