# Supplementary material for: Effectiveness of an intervention designed to optimize statins use: a primary prevention randomized clinical trial
Source: BMC Fam Pract. 2014 Jul 15;15:135. doi: 10.1186/1471-2296-15-135 (PMC4112648; doi:10.1186/1471-2296-15-135)

Additional file **3. Framingham-REGICOR adapted risk charts, simplified into four risk categories based on event concentration data and the 10-year risk cutoff values proposed by experts from various autonomous communities in Spain. HDL: high-density lipoproteins.**


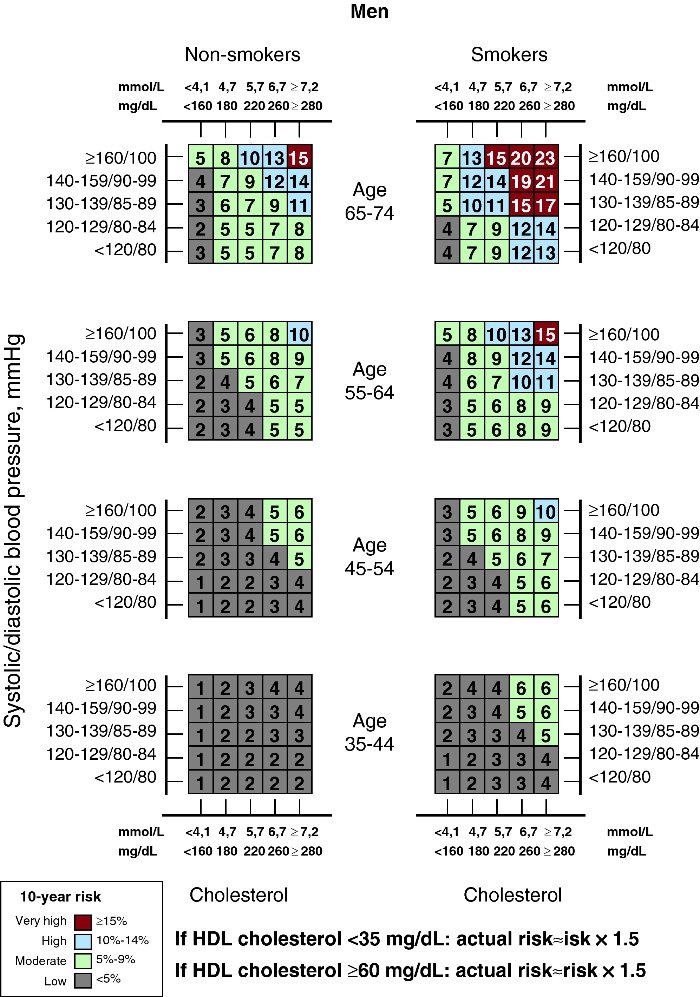


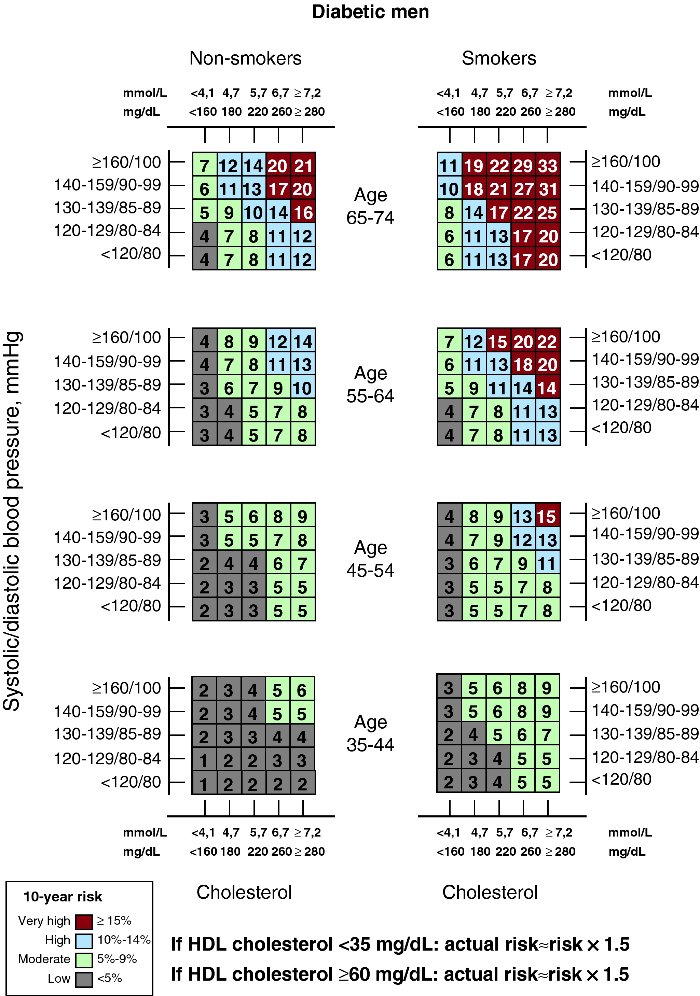


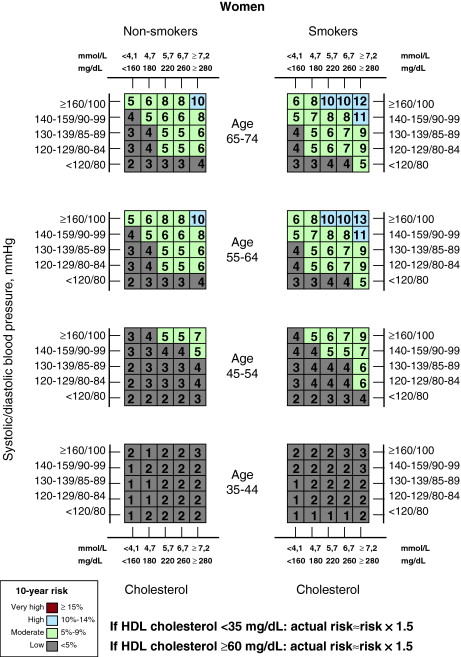


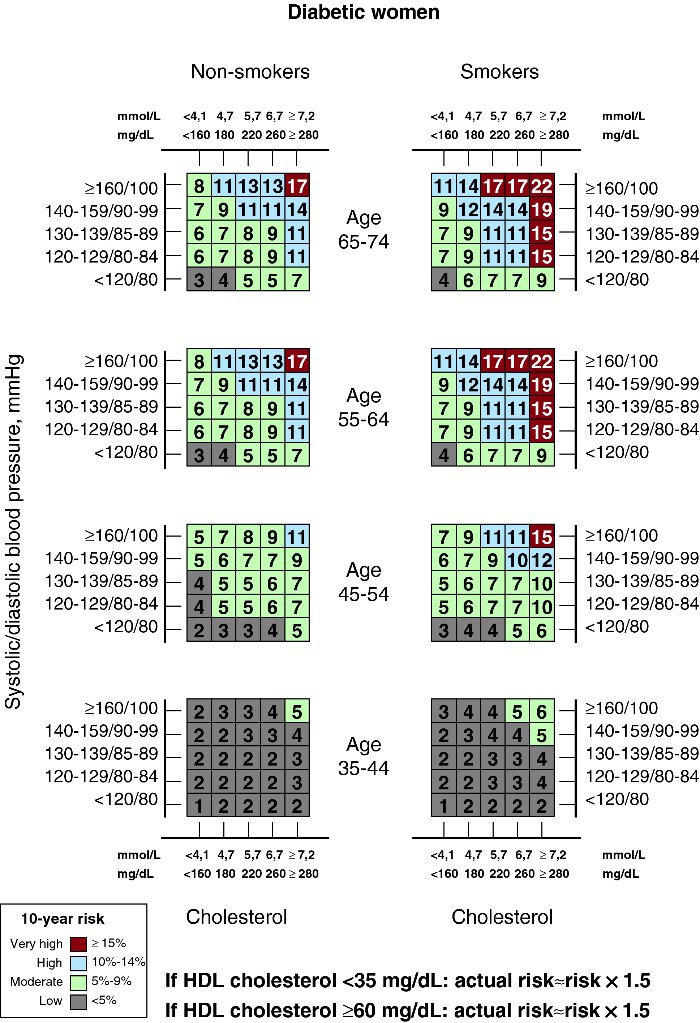

Supplement: Additional file 3 — Framingham-REGICOR adapted risk charts, simplified into four risk categories based on event concentration data and the 10-year risk cutoff values proposed by experts from various autonomous communities in Spain. [file 1471-2296-15-135-S3.docx]
